# Locally placed nanoscale gold islands film within a TiO_2_ photoanode for enhanced plasmon light absorption in dye sensitized solar cells

**DOI:** 10.1186/s40580-016-0093-7

**Published:** 2016-12-07

**Authors:** Taeheon Kim, Yogeenth Kumaresan, Sung Jun Cho, Chang-Lyoul Lee, Heon Lee, Gun Young Jung

**Affiliations:** 1 School of Materials Science and Engineering, Gwangju Institute of Science and Technology (GIST), Gwangju, 61005 Republic of Korea; 2 Advanced Photonics Research Institute (APRI), Gwangju Institute of Science and Technology (GIST), Gwangju, 61005 Republic of Korea; 3 Department of Materials Science and Engineering, Korea University, Seoul, 02841 Republic of Korea

**Keywords:** Dye-sensitized solar cell, Surface plasmon resonance, Photoanode, Absorption enhancement

## Abstract

As metal nanostructures demonstrated extraordinary plasmon resonance, their optical characteristics have widely been investigated in photo-electronic applications. However, there has been no clear demonstration on the location effect of plasmonic metal layer within the photoanode on both optical characteristics and photovoltaic performances. In this research, the gold (Au) nano-islands (NIs) film was embedded at different positions within the TiO_2_ nanoparticulate photoanode in dye-sensitized solar cells (DSSC) to check the effect of plasmon resonance location on the device performance; at the top, in the middle, at the bottom of the TiO_2_ photoanode, and also at all the three positions. The Au NIs were fabricated by annealing a Au thin film at 550 °C. The DSSC having the Au NIs-embedded TiO_2_ photoanode exhibited an increase in short circuit currents (J_sc_) and power conversion efficiency (PCE) owing to the plasmon resonance absorption. Thus, the PCE was increased from 5.92% (reference: only TiO_2_ photoanode) to 6.52% when the Au NIs film was solely positioned at the bottom, in the middle or at the top of TiO_2_ film. When the Au NIs films were placed at all the three positions, the J_sc_ was increased by 16% compared to the reference cell, and consequently the PCE was further increased to 7.01%.

## Background

Many researchers are focusing on the plasmon resonance phenomenon due to their strong absorption and scattering effect [1]. Recently, various nanostructures, including nanoparticles, nanoislands, nanorods, and nanoflowers, have drawn a great attention due to their exceptional surface plasmon resonance phenomenon [2–6]. Various lithographic techniques were employed to design plasmon nanostructures with a controlled size, shape, and arrangement for the surface-enhanced Raman scattering in the field of chemical and biosensors [7]. Among those plasmon nanostructures, nanoparticles have the most effective localized surface plasmon resonance absorption enhancement at visible wavelengths, which can be utilized in energy harvesting, photocatalyst, solar cells or water splitting [8–10]. Furthermore, it has been extensively reported that the plasmon resonance in noble metal nanoparticles can enhance the light trapping within a photovoltaic medium in dye-sensitized solar cells (DSSC) [11].

Dye-sensitized solar cells was firstly demonstrated by Gratzel in 1991 [12], which exhibited plenty of advantages such as its transparency, flexibility, low cost and easy fabrication process. DSSC is composed of three parts; photoanode, electrolyte and counter electrode, among which, the dyes adsorbed within a semiconducting photoanode layer absorb photons and generate electron–hole pairs. The efficiency of DSSC increases with the formation of more electron–hole pairs. It is well known that the TiO_2_ nanoparticulate layer mixed with metallic nanoparticles showed a higher light absorbance due to the localized surface plasmon resonance around the surface of metal nanoparticles, and more electron–hole pairs were generated [13].

Generally, solid thin films are thermodynamically unstable and easy to be transformed into more stable shapes when heated below their melting temperature due to the solid state thermal dewetting phenomenon [14, 15]. This phenomenon occurs to reduce the surface energy of thin film and interfacial energy between the thin film and the substrate. Therefore, while annealing the metal thin film such as gold (Au), Au nanoislands (NIs) film was formed, which revealed plasmon resonance phenomenon at a specific wavelength depending on the island sizes and shapes [16, 17]. To utilize the plasmon resonance phenomenon for enhancing the DSSC efficiency, researchers have incorporated the Au NIs into the TiO_2_ semiconducting layer in the photoanode. However, within our knowledge, there has been no clear demonstration on the location effect of plasmonic metal layer within the photoanode on both optical characteristics and photovoltaic performance.

In this research, we fabricated four different configurations of Au NIs film-embedded TiO_2_ photoanode; solely located at the top, in the middle or at the bottom, and combined at all the three positions, and their plasmon resonance properties were then studied. The size and morphology of Au NIs were optimized by varying the initial Au film thickness. Furthermore, DSSCs having the four different photoanodes were fabricated to study the effect of plasmon resonance location on the DSSC performance.

## Results and discussion

### Morphology of Au NIs

**Fig. 1:**
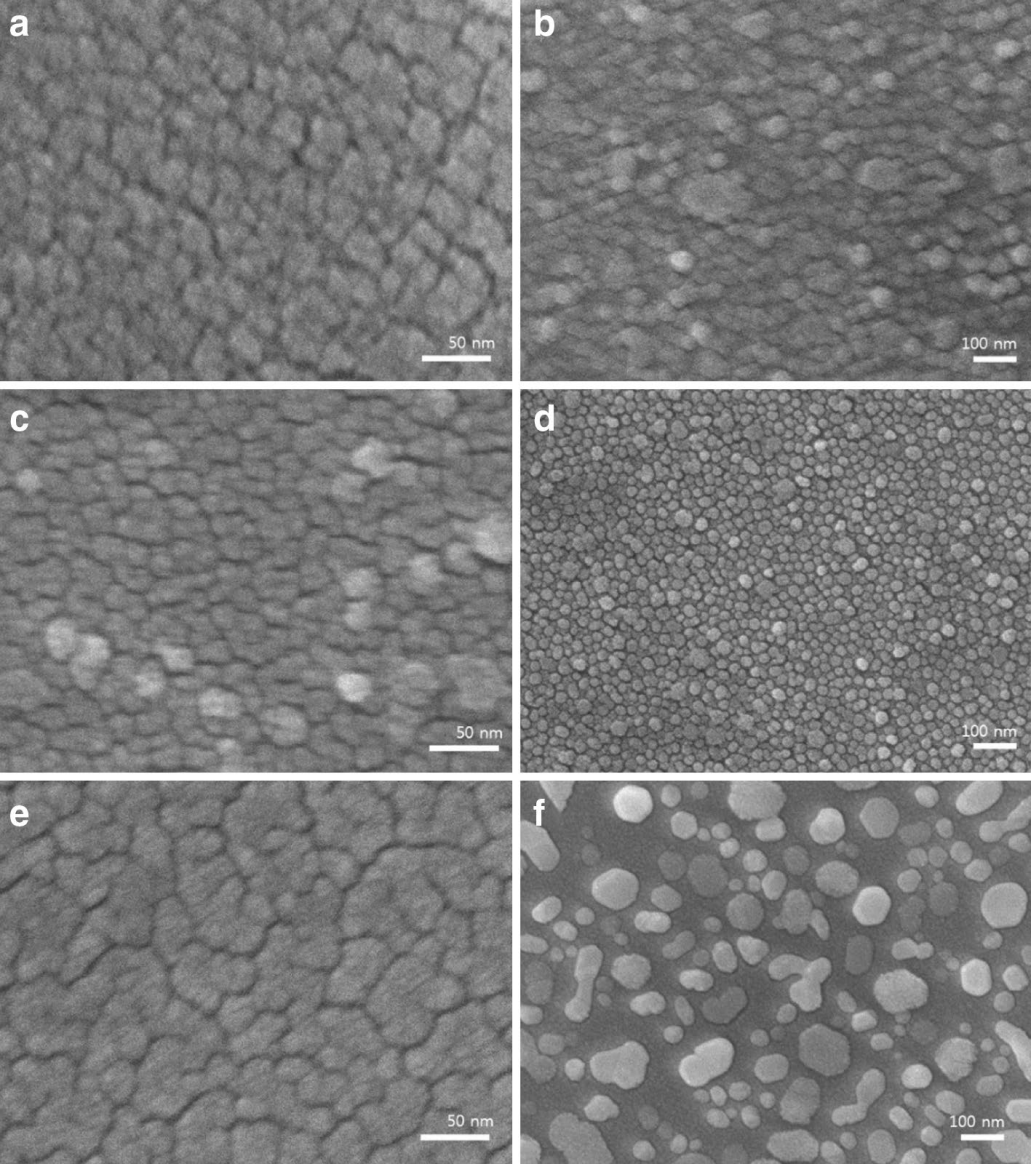
SEM images of Au film and Au NIs; as-deposited Au film with a thickness of **a** 2 nm, **c** 4 nm, and **e** 8 nm: Au NIs after annealing the Au film with a thickness of **b** 2 nm, **d** 4 nm, and **f** 8 nm at 500 °C for 1 h

**Fig. 2:**
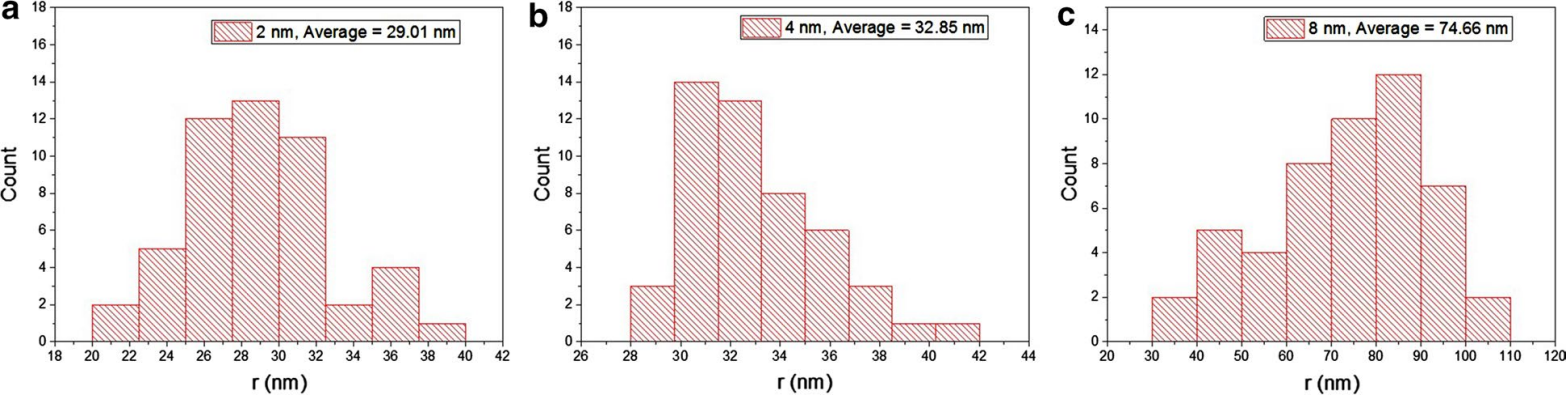
Histogram of Au NIs diameter after annealing the Au film with a thickness of **a** 2 nm, **b** 4 nm, and **c** 8 nm at 500 °C for 1 h

2, 4 and 8 nm thick Au thin films were deposited on a glass substrate by electron-beam evaporator and their morphologies were characterized by using a field emission scanning electron microscopy (FE-SEM, JEOL 2010F) as shown in the left side of Fig. 1, respectively. The corresponding Au NIs film were formed after annealing at 550 °C as shown in the right side of Fig. 1 (detailed in the “Experimental details” section). The size and shape of the Au NIs were different depending on the initial film thickness. The size distribution of the Au NIs are shown in Fig. 2 and the average size and its standard deviation from around 50 Au NIs per sample for statistics are listed in Table 1. The as-evaporated film at such a thin thickness was not smooth but cracked, and the grains were bigger with the initial film thickness. During the annealing, the Au grains separated at the grain boundaries and aggregated to form the Au NIs. The average size of Au NIs increased with the initial thickness. Round-type Au NIs with a few nm gap between them were produced after annealing with a film thickness of less than 4 nm. However, faceted Au NIs were developed in the case of 8 nm thick sample, indicating that the initially larger Au grains aggregated to form a thermodynamically stable faceted island with various a large gap between them (a few tens of nm to over 100 nm).

**Table 1:** Average diameter and its standard deviation of the Au NIs after annealing the Au film with a thickness of 2, 4 and 8 nm at 500 °C for 1 h

Initial Au film thickness (nm)	2 nm	4 nm	8 nm
Average diameter	29.01	32.85	74.66
Standard deviation	3.98	2.95	18.11

### Optical characterization of Au NIs

**Fig. 3:**
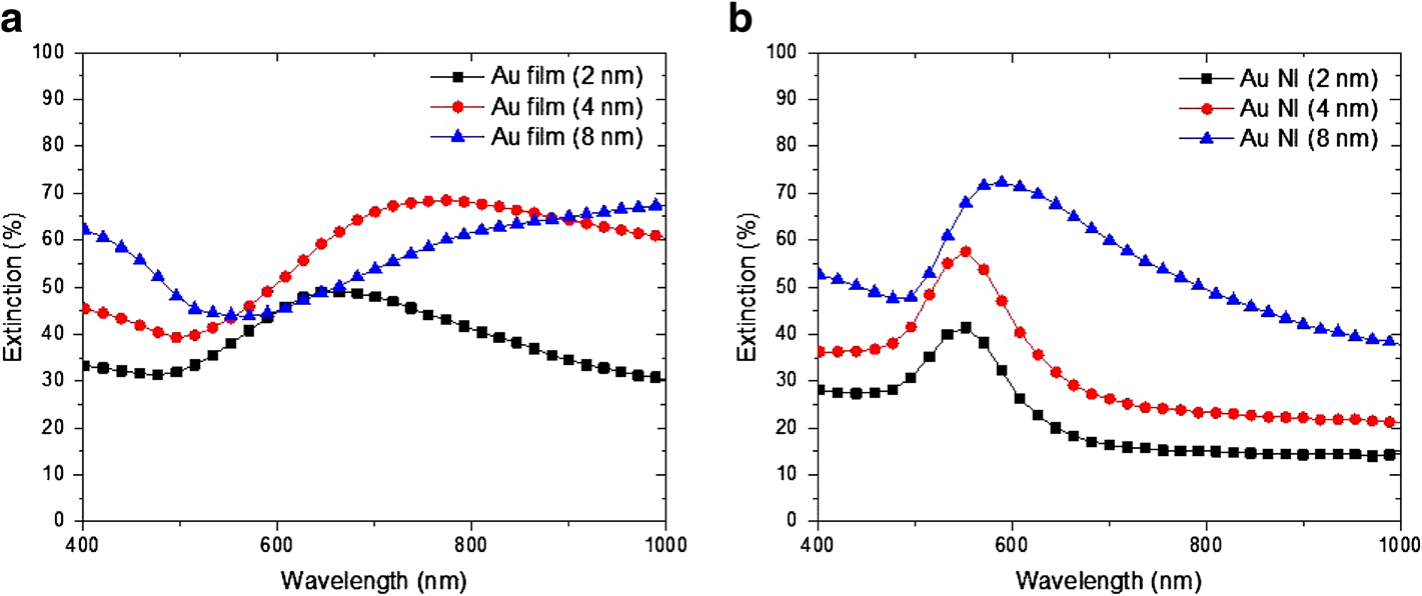
Absorbance spectra of **a** the as-deposited Au films at different thicknesses and **b** the Au NIs films after annealing the respective Au film with a corresponding initial thickness

The optical properties of Au NIs film were measured by UV–Vis. spectroscopy (AvaSpec-UL2048L-USB2 spectrometer, Jinyoung tech Inc.). The absorbance spectra of the 2, 4 and 8 nm thick Au films are compared in Fig. 3a with a reference to the bare glass substrate, in which the absorption peak shifts from 645 for 2 nm sample to 773 for 4 nm sample and even to infrared for 8 nm sample with extinction values ranged from 35 to 75%. After annealing at 550 °C for 1 h, the absorption peak was blue-shifted in all the samples; a plasmon resonance peak arising from the Au NIs was observed at 550 nm for the 2 and 4 nm samples and at 590 nm for the 8 nm sample as shown in Fig. 3b. This plasmon resonance absorption peak is well matched with the absorption peak (~550 nm) of N719 material [18], which has been commonly used as a dye material in DSSCs to generate electron–hole pairs by absorbing the Sun light. Therefore, the Au NIs film was incorporated into the TiO_2_ nanoparticulate photoanode in DSSCs, which was adsorbed by the N719 dye molecules. It was expected to enhance the light absorption of dyes owing to the plasmon resonance absorption around the Au NIs, generating more electron–hole pairs and thus more photocurrents.

The Au NIs film within the TiO_2_ nanoparticulate layer should be transparent to the light concurrently so that the penetrated light can excite the dyes adsorbed within the rest TiO_2_ film. Considering the plasmon resonance absorption peak at 550 nm and the appropriate transparency, the 4 nm thick Au film was chosen to fabricate Au NIs film-incorporated TiO_2_ photoanode in the following DSSC fabrication with the four different configurations; solely located at the top, in the middle, at the bottom, and combined at all the three positions within the TiO_2_ film, which are named hereafter as top, middle, bottom and all configuration sample, respectively (detailed in the “Experimental details” section). For the DSSC fabrication, the TiO_2_ nanoparticulate paste was coated onto a fluorine doped tin oxide (FTO) glass by doctor-blade method.

**Fig. 4:**
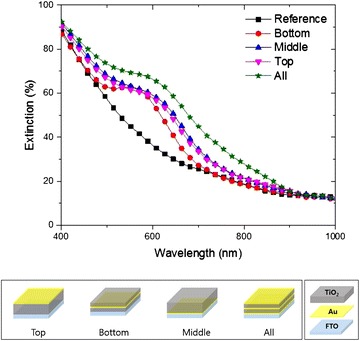
Absorbance spectra of Au NIs-incorporated TiO_2_ photoanodes at different positions (*top*, *bottom*, *middle*, and *all* configurations)

Figure 4 compares the absorbance spectra from the 5 different photoanodes including the reference FTO glass. The FTO glass itself had a monotonous decrease in absorbance in the visible wavelengths. Meanwhile, the other Au NIs film-incorporated photoanodes had a higher extinction values in the entire visible wavelengths with a shoulder around 600 nm, which was arising from the plasmon resonance absorption at the Au NIs. The all configuration photoanode had the highest extinction value among the samples. Meanwhile, the other Au NIs film-incorporated samples showed the similar light absorption spectra. Interestingly, the plasmon resonance peak was shifted from 550 nm for the only Au NIs on top of a glass substrate (Fig. 3b) to 600 nm for the Au NIs-incorporated TiO_2_ nanoparticulate film on a FTO substrate. It was reported that the coating of silver islands film with a dielectric medium of TiO_2_ shifted the plasmon resonance peak towards the red [19, 20]. As our Au NIs were coated and surrounded by the dielectric TiO_2_ medium, the plasmon resonance peak was accordingly red-shifted.

### Comparison of DSSC performance

**Fig. 5:**
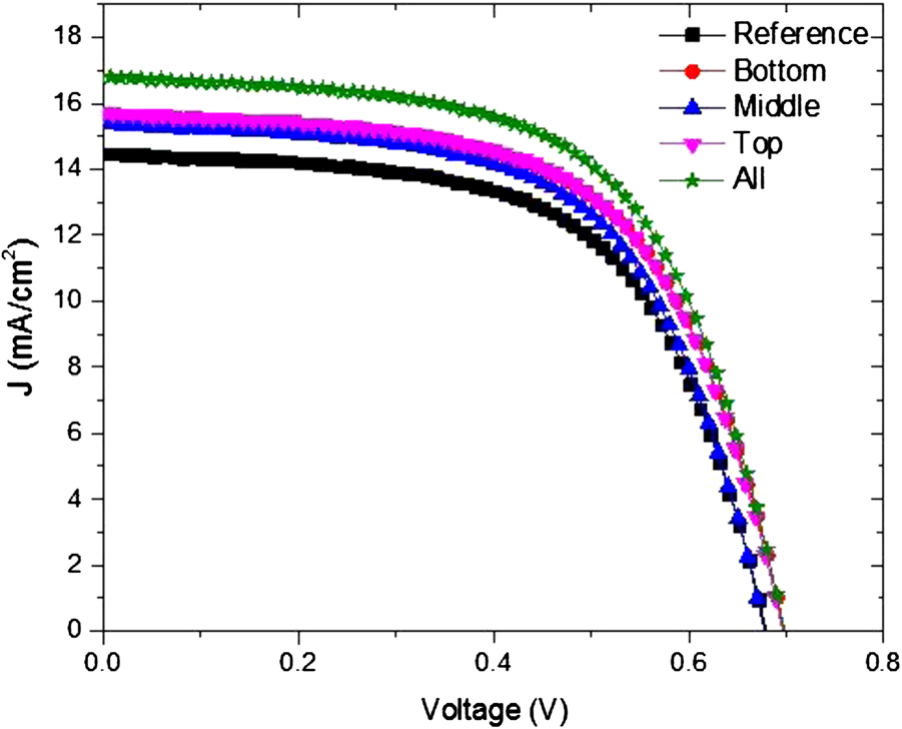
*J*–*V curves* of DSSCs having the different photoanodes

A set of DSSCs having the different photoanodes as mentioned before were fabricated and the solar cells were measured to check the effect of Au NIs location on the device performance. The DSSCs were measured by using Keithley 2400 source-meter under one sun condition (AM 1.5G, 100 mW/cm^2^, SANEI solar simulator, Class A) to obtain current density–voltage curve (J-V curve) including the open circuit voltage (V_oc_), short circuit current (J_sc_), fill factor (FF), and power conversion efficiency (PCE). Figure 5 compares the J-V curves of the five DSSCs and the solar cell performance parameters are given in Table 2. The reference cell without the Au NIs had the poorest solar cell performance and All configuration DSSC had the highest power conversion efficiency among the samples. The J_sc_, V_oc_, FF and PCE values of the reference cell is 14.4 mA/cm^2^, 0.67 V, 0.61 and 5.92%, respectively. Compared to the reference cell, the bottom, middle and top configuration DSSCs had an increased J_sc_ by ~8% and the J_sc_ of all configuration DSSC was increased by 16% from 14.4 to 16.8 mA/cm^2^ along with the improved PCE by 18% from 5.92 to 7.01%. The solar cell performances are well in coincidence with the tendency of plasmon resonance absorption increment among the samples (Fig. 4), in which the all configuration photoanode has the highest absorption, whereas the other three Au NIs-incorporated photoanodes have the similar absorption spectra in the visible wavelengths. Therefore, the increase in J_sc_ is ascribed to the plasmon resonance-induced light absorption enhancement. The V_oc_ was almost similar among the samples regardless of the location of Au NIs film within the TiO_2_ film.

**Table 2:** DSSC performances (V_oc_, J_sc_, FF, and PCE) of DSSCs having the different photoanodes

	V_oc_ (V)	J_sc_ (mA/cm^2^)	FF	PCE (%)
Reference	0.67	14.4	0.61	5.92
Bottom	0.69	15.5	0.60	6.52
Middle	0.67	15.3	0.61	6.37
Top	0.68	15.6	0.61	6.49
All	0.69	16.8	0.60	7.01

**Fig. 6:**
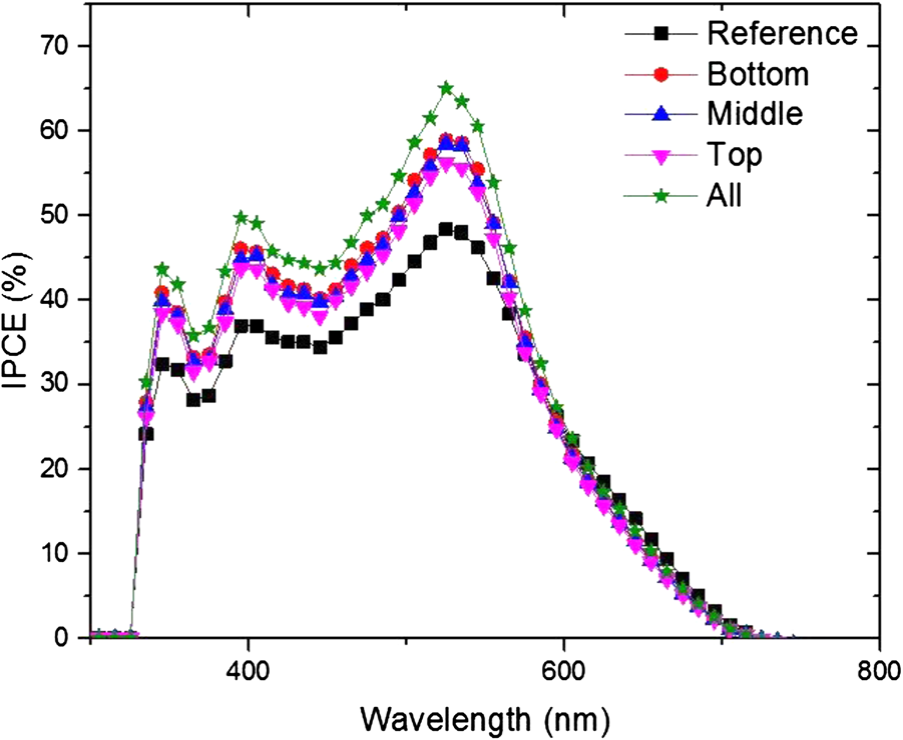
*IPCE curves* of DSSCs having the different photoanodes

External quantum efficiency (EQE) was measured at 300–700 nm by incident photon-to-current efficiency (IPCE) measurement. In accordance with both the optical spectra and J-V characteristics among the samples, IPCE curves showed the same tendency among the samples as shown in Fig. 6. The all configuration DSSC had the highest EQE value in each wavelength, revealing the best solar cell performance among the samples.

## Conclusions

In summary, the Au NIs film was placed at different positions within a TiO_2_ photoanode to exploit the effect of Au NIs location on the surface plasmon resonance phenomenon. Au NIs were spontaneously generated from an Au thin film after thermal treatment at 550 °C for 1 h and the average size of Au NIs increased with the initial Au film thickness. Au NIs with a diameter of 33 nm in average were produced from a 4 nm thick Au film and revealed a plasmon resonance absorption at 550 nm, which was well matched with the absorption peak of N719 dye material.

The Au NIs film was incorporated at different locations within a TiO_2_ film to generate the bottom, middle, top, and all configuration photoanodes for DSSCs fabrication. The DSSCs having the Au NIs-incorporated photoanode exhibited the higher J_SC_ compared to the reference cell owing to the enhanced plasmon resonance light absorption. The all configuration solar cell had the highest J_sc_ among the cells. Consequently, the PCE was increased from 5.92% for the reference cell to ~6.4% for the single Au NIs film-incorporated cells, and to 7.01% for the all configuration cell. The three single Au NIs film-incorporated photoanodes demonstrated the similar optical properties and solar performances, indicating that there was no specific effect of plasmon resonance location on solar cell performances.

## Experimental details

### Formation of Au NIs

The Au NIs were formed by thermal annealing process of Au thin film. Prior to the deposition of Au thin film, the target substrate such as glass, silicon or fluorine doped tin oxide (FTO, 16 Ω/cm) was cleaned by sonication in acetone, IPA and deionized water for 15 min, respectively, and dried with a nitrogen gun. The thin Au film was deposited by electron beam (e-beam) evaporator on the substrates and/or on top of the TiO_2_ nanoparticulate film coated on the FTO substrate at a different thickness and then annealed at 550 °C for 1 h by using a wind furnace to form the Au NIs.

### Fabrication of photoanodes

Fluorine doped tin oxide glass substrates were used to fabricate the photoanode and the counter electrode in a DSSC. Reference photoanode was fabricated by coating the mesoporous TiO_2_ (TTP-20 N, ENB-T1204051) nanoparticulate paste by doctor blade technique on top of the FTO glass. Immediately, it was baked on a hot plate at 150 °C for 30 min to remove the remaining solvent within the TiO_2_ film and then sintered at 450 °C for 90 min in air atmosphere. After sintering, 12 μm thick TiO_2_ photoanode was prepared in the area of 0.25 cm^2^. As-prepared photoanode was immersed into 0.5 mM N719 dye solution (Ruthenizer 535-bis TBA, Solaronix, Aubonne, Switzerland) in 1:1 (v/v) mixed solution of acetonitirile (ACN) and tert-butanol, for 12 h to adsorb the dye molecules onto the TiO_2_ nanoparticulate film. The photoanode was then rinsed in ethanol to remove excessive dyes and dried in air.

The Au NIs film-incorporated TiO_2_ photoanodes having the Au NIs film positioned at the top, in the middle, at the bottom and at all the three positions within the TiO_2_ nanoparticulate film were fabricated. The top configuration photoanode was fabricated by depositing 4 nm thick Au film on top of the 12 μm TiO_2_/FTO substrate and then annealed to form the Au NIs. The middle configuration photoanode was fabricated by depositing the Au thin film on the 6 μm TiO_2_/FTO substrate and then annealed to form the Au NIs. Then, TiO_2_ paste was again coated on top of the Au NIs and sintered to have another 6 μm thick TiO_2_ film. The bottom configuration photoanode was fabricated by firstly making the Au NIs film on the FTO substrate and then 12 μm thick TiO_2_ nanoparticulate film was placed on it. The all configuration photoanode was fabricated by repeating the necessary processes to have three layers of NIs film within the TiO_2_ photoanode. Each photoanode configuration is illustrated at the bottom of Fig. 4.

### Fabrication of DSSCs

The counter electrode was fabricated by depositing a 20 nm thick platinum on the FTO glass substrate by using e-beam evaporator. Finally, for assembling the DSSC, the fabricated photoanode and counter electrode were attached using a 30 μm thick surlyn spacer (Dupont) and annealed at 90 °C on a hot plate. Before measuring the DSSC performance, the electrolyte, consisted of 0.6 M 1-butyl-3-methylimidaxolium iodide (C6DMI), 0.04 M I_2_, 0.2 M LiI_2_ and 0.5 M tert-butyl pyridine (TBP) in a 1:1 (v/v) mixture of acetonitrile (CAN) and 3-methoxy propiontirile (MPN), was injected into the gap between the photoanode and counter electrode.
